# Taking a look to promoting health and complications' prevention: differences by context

**DOI:** 10.1590/1518-8345.0860.2749

**Published:** 2016-08-08

**Authors:** Rosa Maria de Albuquerque Freire, Maria José Lumini Landeiro, Maria Manuela Ferreira Pereira da Silva Martins, Teresa Martins, Heloísa Helena Ciqueto Peres

**Affiliations:** 1 Doctoral Student, Instituto de Ciências Biomédicas Abel Salazar, Universidade do Porto, Porto, Portugal. Adjunct Professor, Escola Superior de Enfermagem do Porto, Porto, Portugal.; 2 PhD, Professor, Escola Superior de Enfermagem do Porto, Porto, Portugal.; 3 PhD, Full Professor, Escola de Enfermagem, Universidade de São Paulo, São Paulo, SP, Brazil.

**Keywords:** Health Promotion, Complications, Nursing, Nursing Process, Patient-Centered Care

## Abstract

**Objectives:**

to acknowledge and compare the health promotion and complications' prevention practices performed by nurses working in hospital and primary health care contexts.

**Methods:**

descriptive, exploratory and crosscutting study, performed with 474 nurses selected by convenience sampling. It was used a form that encompassed two categories of descriptive statements about quality in the professional exercise of nurses. This study had ethical committee approval.

**Results:**

the nurses' population was mainly women (87,3%) with an average age of 35,5 years. There was more practices of the hospital's nurses related to the identification of potential problems of the patient (p=0.001) and supervision of the activities that put in place the nursing interventions and the activities that they delegate (p=0.003).

**Conclusion:**

the nurses perform health promotion and complications' prevention activities, however not in a systematic fashion and professional practices differ by context. This study is relevant as it may promote the critical consciousness of the nurses about the need of stressing quality practices.

## Introduction

Health promotion and complications' prevention are two descriptive statements of the quality standards for the professional exercise of nurses in Portugal. Nursing care quality standards were established in 2001 by the Portugal Order of Nurses, aiming to improve the services performed by these practitioners, and gaining visibility to the professional group with regard to the role that hey have in society as a whole^1^, at the same time being part of their performance evaluation. 

The World Health Organization (WHO) defined health promotion in 1986 as "the process that trains individuals to get grasp of and improve their own health^2^". In this sense it is understood that individuals need to develop capabilities and competencies to enable them to adapt to the different stages of the vital cycle and to their health/illness processes in an effective way. 

Nurses may help to foster this process. For that end they need to place the patient in the center stage of care and be able to perform a holistic analysis of the person, family groups and community in a way that allows identifying their peculiarities in the realm of health promotion. The patient-centered care demands for the comprehensiveness of the health promotion activities within the nurses' clinical practice, thus being a requisite for their professional practice.

Implementing interventions in the realm of health promotion, directed towards empowering of the patient and developing coping strategies, may help to manage the weaknesses that a chronic disease carries in itself. These interventions are even more remarkable in the situation when there are low availability of psychosocial resources, such as those of social isolation and loneliness, low self-esteem, feeling unsafe, exhaustion, depression and low socioeconomic level^3^, conditions that are frequently associated to chronic disease conditions.

The health promotion interventions may be of the individual, community, organizational or governmental types. The individual-level interventions are directed to knowledge, attitudes and/or behaviors. The organizational, community or environmental interventions are focused on policies, programs, facilities or resources; and the governmental level ones act on the legislation, regulation and execution of the health policies^4^. The intervention areas may be classified by health promotion levels: the basic level includes the primary, secondary and tertiary illness preventative measures, communication, health information for all educational levels and social marketing campaigns and behavioral change campaigns; the intermediate level encompasses health education and training, personal competencies to manage their own health and well-being, knowledge and understanding about what fosters good health, supportive environments, community development, partnerships, commitment, training and community actions; the upper level encompasses the infrastructure and change systems, public health policies, regulation and legislation, reorienting the health system, organizational change and inter-sectorial collaboration^5^. Thus, health promotion becomes actually the aim of the attention of the whole society. 

In spite of this, the health promotion concept is oftentimes mistaken with the complications' prevention, being the latter related to the potential problems of the patient and to the risks that are intrinsic or extrinsic to the individual, problems that frequently demand the nurses' interventions for their control.

In this perspective and considering that the Portuguese nurses have the duty to implement in their clinical activities the interventions as proposed by the quality standards that were developed by the Portugal Order of Nurses, it was proposed to develop this pioneering study with the aim to know and compare the nurses' practices in two organizational contexts, primary health care and hospital. For this end it was used a guiding question: to know "if there are significant differences between the nurses' practices in their work in a hospital or in primary care units, in the realms of health promotion and complications' prevention" 

## Methods

Exploratory, descriptive and crosscutting study using a quantitative approach, approved by the Ethics in Health Committees and by the Administrative Boards of the institutions where the study was developed, according to the verdicts 159/13 of July 25^th^ 2013 and 68/13 of February 14^th^ 2014, observing the ethical principles inherent to research as defined by Law 21/2014 of Portugal of April 16^th(^ ^6^ ^).^

The sample was made up with 474 nurses exercising in the care area: 235 nurses pertaining to a central hospital in the North of Portugal and 239 nurses working as practitioners in a Group of Health Centers (ACeS^**)^ of the center region of Portugal. Based in the previous partnership for in-service educational activities that the researchers had in the past with those institutions where the study was developed, the convenience non-probabilistic sampling method was adopted. The inclusion criteria were linked to the availability and interest in answering to the survey form, decision that was preceded by information about the objectives and aims of the study, and the agreement in freely participating of the research. The Free and Informed Consent Form was handed out at the same moment with the survey form and with an envelope to return the survey form after completion. Previously to the data collection, members of the research team personally contacted each one of the research subjects. The return of the survey form was done in an envelope that was sealed by the respective chief of nursing.

Data collection was done in the hospital context from September to November 2013 and in the Primary Care context from March to May 2014.

The survey form was organized in two parts. The first had five questions that aimed to identify the demographic and professional characteristics of the participant: sex, age, academic achievement, time of professional practice and time of practice in the present site. The second part added two quality standards for the nurses' professional practice: health promotion and complications' prevention and their respective descriptive statements, as formulated by the Portugal Order of Nurses^1^ and publicly available without restrictions. These descriptive statements were questions that were previously analyzed by specialists in each topic, to check for clarity, understanding, language and pertinence. 

The survey form had three questions related to health promotion and seven questions to complications' prevention. These questions are measured in a four points Likert scale, ranging from: 1 - never; 2 - seldom (less than half of the time); 3 - sometimes (more that half of the time); 4 - always.

The analysis of data for describing the demographic and professional profile was done through descriptive statistics through absolute and relative frequency distribution, and for continuous variables the central trend and dispersion measures were used. The comparison between groups was done through inferential statistic using Student's t-test in the continuous variables and Pearson Chi-squared test (χ2) for the categorical variables for a significance level of p < 0.05.

## Results 

From the 474 nurses participating in the study, 49,6% (235) were from the hospital and 50,4% (239) from the primary health care: 87,3% were female and 12,7% male; ages ranged between 24 and 60 years old; time in the profession and in the present post ranged between 1 and 38 years; 68,1% had only degrees in nursing and 31,9% had also post- graduate courses. The participants coming from the hospital had an age average of 35,5±8,2 y.o., average of practicing the profession of 12,8±8,1 years and average of performing as professionals in the present site of 8,1±7,1 years. The participants from the primary health care setting had an age average of 35,5±9,1 y.o., average of practicing the profession of 8,4±6,5 years and average of performing as professionals in the present site of 2,1±0,9 years. Table 1 presents the differences between the participants of both contexts regarding demographic and professional characteristics. 

**Table 1:** Frequency distribution of nurses in both contexts according to gender, age, and academic achievement, lifelong time of practice and time of practice in present site. Hospital (n=235) and ACeS (n=239). Porto and Coimbra, Portugal, 2013-2014.

Variables		Hospital (n=235)		ACeS^†^ (n=239)		Total		p
		n	%	n	%	n	%	
Sex								0.001*
	Males	42	17,9	18	7,5	60	12,7	
	Females	193	82,1	221	92,5	414	87,3	
Age groups								
	21-30	85	36,2	80	33,5	165	34,8	0.026*
	31-40	91	38,7	99	41,4	190	40,1	
	41-50	45	19,1	30	12,6	75	15,8	
	51-60	14	6	30	12,6	44	9,3	
Academic achievement								<0.001*
	Degree in nursing	170	72,3	65	27,2	235	49,6	
	Post-graduate	65	27,7	174	72,8	239	50,4	
Lifelong time of practice (years)								<0.001*
	1-10	116	49,4	172	72	288	60,8	
	11-20	79	33,6	52	21,8	131	27,6	
	21-30	31	13,2	12	5	43	9,1	
	31-40	9	3,8	3	1,3	12	2,5	
Time of practice in present site (years)								<0.001*
	1-10	168	71,5	239	100	407	85,9	
	11-20	51	21,7	0	0	51	10,8	
	21-30	12	5,1	0	0	12	2,5	
	31-40	4	1,7	0	0	4	0,8	

*p <0.05

†ACeS: Group of Health Units

The groups show differences regarding the socio-demographic variables, and those differences are statistically significant.

Table 2 presents the nurses' practices referred to the quality standards under analysis looking at the sample as a whole:

**Table 2:** Frequency distribution of nurses by the quality standards for professional practice in health promotion and complications' prevention and their respective descriptive statements (n =474). Porto and Coimbra, Portugal, 2013-2014.

Variables		Never		Seldom		Sometimes		Always		p
		n	%	n	%	n	%	n	%	
Health promotion										
	Nurses identify the health situations of the population and the patients, family and community resources	1	0,2	51	10,8	294	62	128	27	<0.001^*^
	Nurses use the opportunity of hospitalization to promote healthy lifestyles	1	0,2	47	9,9	224	47,3	202	42,6	<0.001^*^
	Nurses provide information fostering cognitive learning and new capabilities for the patient	-		31	6,5	249	52,5	194	40,9	<0.001^*^
Complications' prevention										
	Nurses identify the potential problems of the patient	-		15	3,2	257	54,2	202	42,6	<0.001^*^
	Nurses prescribe and implement interventions geared towards the complications' prevention	-		21	4,4	235	49,6	218	46	<0.001^*^
	Nurses assess the interventions that will help to avoid problems or minimize undesirable effects	-		41	8,6	221	46,6	212	44,7	<0.001^*^
	Nurses show stringent scientific and technical stance in implementing nursing interventions	-		26	5,5	232	48,9	216	45,6	<0.001^*^
	Nurses refer problematic cases to other professionals according to social mandates	1	0,2	39	8,2	228	48,1	206	43,5	<0.001^*^
	Nurses supervise the activities that put in place nursing interventions and those that they delegate	-		46	9,7	250	52,7	178	37,6	<0.001^*^
	Nurses show responsibility for their decisions, for their acts and for those that they delegate	-		11	2,3	55	32,7	308	65	<0.001^*^

*p <0.05.

The answers of the interviewees are statistically significant for all questions.

The practices of nurses regarding the quality standard health promotion, analyzed by health institution are presented in Table 3

**Table 3:** Frequency distribution of nurses of both contexts by the quality standards for professional practice in health promotion and their respective descriptive statements by groups Hospital (n=235) and ACeS (n=239). Porto and Coimbra, Portugal, 2013-2014.

Variables		Never	Seldom			Sometimes			Always		p
		n	%		n	%	n	%	n	%	
Health promotion											
	Nurses identify the health situations of the population and the patients, family and community resources	Hospital	1	0,4	23	9,8	154	65,5	57	24,3	0.301
		ACeS^†^	0	0	28	11,7	140	58,6	71	29,7	
	Nurses use the opportunity of hospitalization to promote healthy lifestyles	Hospital	0	0	30	12,8	129	54,9	76	32,3	<0.001^*^
		ACeS^†^	1	0.4	17	7,1	95	39,7	126	52,7	
	Nurses provide information fostering cognitive learning and new capabilities for the patient	Hospital	0	0	18	7,7	132	56,2	85	36,2	0.098
		ACeS^†^	0	0	13	5,4	117	49	109	45,6	

*p <0.05

†Group of Health Units.

The nurses' practices regarding the quality standard complications' prevention, analyzed by health organizations are shown in Table 4.

**Table 4:** Frequency distribution of nurses of both contexts by the quality standards for professional practice in complications' prevention and their respective descriptive statements by groups Hospital (n=235) and ACeS (n=239). Porto and Coimbra, Portugal, 2013-2014.

Variables		Never	Seldom			Sometimes			Always		p
		n	%		n	%	n	%	n	%	
Complications' prevention											
	Nurses identify the potential problems of the patients	Hospital	0	0	4	1,7	112	47,7	119	50,6	0.001^*^
		ACeS^†^	0	0	11	4,6	145	60,7	83	34,7	
	Nurses prescribe and implement interventions geared towards the prevention of complications	Hospital	0	0	12	5,1	107	45,5	116	49,4	0.205
		ACeS^†^	0	0	9	3,7	128	53,6	102	42,7	
	Nurses assess the interventions that will help to avoid problems or minimize undesirable effects	Hospital	0	0	14	6	108	46	113	48	0.077
		ACeS^†^	0	0	27	11,3	113	47,3	99	41,4	
	Nurses show stringent scientific and technical stance in implementing nursing interventions. enfermagem	Hospital	0	0	11	4,7	114	48,5	110	46,8	0.696
		ACeS^†^	0	0	15	6,3	118	49,4	106	44,3	
	Nurses refer problematic cases to other professionals according to social mandates	Hospital	1	0,4	15	6,4	127	54	92	39,1	0.039^*^
		ACeS^†^	0	0	24	10	101	42,3	114	47,7	
	Nurses supervise the activities that put in place nursing interventions and those that they delegate	Hospital	0	0	12	5,1	126	53,6	97	41,3	0.003^*^
		ACeS^†^	0	0	34	14,2	124	51,9	81	33,9	
	Nurses show responsibility for their decisions, for their acts and for those that they delegate	Hospital	0	0	4	1,7	76	32,3	155	66	0.652
		ACeS^†^	0	0	7	2,9	79	33,1	153	64	

*p <0.05

†Group of Health Units

When comparing the nurses' practices in both health institutions, we can observe statistically significant differences at the standard of quality for health promotion in the statement "Nurses use the opportunity of hospitalization to promote healthy lifestyles" (p<0.001) and of the quality standard for complications' prevention in the statements "Nurses identify the potential problems of the patients" (p=0.001), "Nurses refer problematic cases to other professionals according to social mandates" (p=0.039), "Nurses supervise the activities that put in place nursing interventions and those that they delegate" (p=0.003).

## Discussion 

The largest share of nurses that were part of this study are women in the sample as a whole and also in the population of each institution. This is also pointed out as a national and international fact, as the prevalence of females in the Nursing profession is still true nowadays and for a long time^7^.

Statistic differences were found in nurses in both contexts, hospital and primary care, related to socio-demographic profile and also to the practices related to health promotion and complications' prevention.

The prevalence of male nurses is larger in the hospital and the female nurses are more prevalent in the primary health care.

The population in this study is young, mainly under 40 years in both institutions. However in the older age groups, nurses in primary care are double in proportion than those in the hospital.

In primary health care, most nurses have less than 10 years of lifelong practice of nursing, they all have less than 10 years of practice in the present site and the majority has post-graduate diplomas. This situation may be a result of the re-organization of primary health care that happened in the last decade in Portugal, when the ACeS, Group of Health Units and the Family Health Units were installed, opening employment opportunities in primary care both for recently graduated and post-graduated nurses.

With regard to the category of the quality standard for health promotion, in the whole sample there was found that majority of nurses identify the population's health situations and the resources of the patient, family and community, and use the opportunity of the hospitalization to promote healthy lifestyles and to provide information that will foster the cognitive learning and new capabilities for the patient. Not withstanding this fact, they do not perform these practices in a systematic fashion, once that is observed that the prevalence of the category "sometimes" is larger than the category "always". 

In this quality standard, there was also a significant difference between the practices of nurses in the two contexts, in relation to the promotion of healthy lifestyles that is a more permanent practice in practitioners acting in primary care in this study. This is also seen in other contexts. A study in Australia reveals that even having evidence that nurses are efficient making interventions for health promotion, it is still needed to enlarge their competencies and expand their interventions to other contexts beyond primary care, as they have a considerable potential in this field^8^. 

In the same fashion, in the category of the quality standard for complications' prevention, the most cited answer is "sometimes", excepted the statement "Nurses show responsibility for their decisions, for their acts and for those that they delegate". Nurses in a non-systematic way: identify the potential problems of the patients; prescribe and implement interventions geared towards the complications' prevention, assess the interventions that will help to avoid problems or minimize undesirable effects, show stringent scientific and technical stance in implementing nursing interventions, refer problematic cases to other professionals according to social mandates, and supervise the activities of direct or indirect care.

In the statements regarding the quality standard for complications' prevention, it is implicit the nursing process for practice systemization, and the findings confirm some weakness in its use.

The nursing process seen as a systematic and dynamic way of delivering care, centered in the patient, is geared towards a result, with evidence of being cost-beneficial and have by foundations the fact that planning and implementation of the nursing interventions should not be dissociated from the values, interests and desires of individuals, families and communities^9^. It is thus a tool to facilitate the humane care and quality of the professional practice to be performed by the nurses in their clinical practice. This imperative places the patients in the center stage of care, promoting positive results in their satisfaction and heath^10^.

When comparing the participants' practices in both contexts it was found that nurses in primary care develop strategies to promote healthy lifestyles and send problematic situations to be seen by other professionals, according to social mandates, in a more systematic fashion than the hospital's nurses. On the other hand, hospital's nurses identify potential problems of the patient and supervise direct and indirect activities of care in a more careful way than the primary care nurses.

 These results show that nurses in primary care are more comfortable using the development and community extension models in their practices, based in the social framework of health, than the hospital's nurses. This confirms the findings of other studies that reveal that health promotion activities are more strongly performed by primary health care nurses^11^.

The physical aspects of illness have guided the clinical practices in the hospital context and in this milieu, nurses not only are not associating health promotion to their practices, but they also consider it a second level priority^12^, hampering the development of health promotion in hospital environments^13^, even though it is considered a crosscutting, multi and inter-disciplinary strategy. 

The request to use the principles of health promotion in all organizations, including hospitals^14^, pre-supposes to consider this environment not only as a curative or illness-preventative context, but also as a factor promoting healthy life^15^, oriented towards training the patients to be active agents in the process of managing their health and illness. With better the adherence of the patients to their health processes, the safety will be improved, better health results will be achieved, costs will fall^16^, effectiveness of interventions will be achieved, as well as quality of life and health expectancy that go beyond the economic benefits for the client, the family, the society as a whole, and the health system. 

Supply of preventative health services may rise health levels and prevent illnesses^17^. Systemizing nursing practices may help to make effective the requisites needed to implement the interventions that go hand in hand with the implementation to the complications' prevention. 

On the other hand, the programs for health promotion may foster self-protective behaviors, responsibility for own health, community participation and adoption of healthy lifestyles^18^. Nurses have a privileged role in the implementation of health promoting interventions^19^ independently from the context where they practice. However, they have sparse proactivity in regard to the adoption of health promotion and self care measures. The care is delivered in a fragmented manner^20^ leaving doubts about its effectiveness, low motivation and lack of training^21^.

Considering that health promotion is associated with the universal principles of Nursing, nurses should have knowledge, competency and skills to articulate its actions in their clinical practice, being this practice in the hospital or primary care contexts.^22^.

Under this perspective, it is needed to re-structure professional practices, implying previously a change in the mindset that substitutes the bio-medical paradigm approach by the paradigm that generates health, a process that needs individual adaptation and professional competency development to foster knowledge, skills, attitudes and consciousness in the patients as pre-requisites for efficient self-care.

## Conclusion

The results of this study allowed the understanding of how the Portuguese nurses articulate in their clinical practices the interventions regarding to quality standards, health promotion and complications' prevention. Not withstanding this finding, this process is not performed in a systematic manner and professional practices diverge according with the context. 

Nurses need to involve themselves deeper with the practices that put in operation the quality standards, not just because they need to conform to professional norms linked to their performance quality, but also because they may fulfill higher purposes associated to raising the visibility of the role of nurses in society. 

We need to remark, as a study limitation, the fact that we examined just two different institutional contexts and two categories of standards for quality of nursing. For a wider vision of the Portuguese practitioners ownership of the quality standards of nursing care as stated by the Portugal Order of Nurses, it is suggested to develop larger studies, at a national level and approaching all the categories of the quality standards that are inherent to nurses' professional practice.

## References

[1] Ordem dos Enfermeiros (2001). Divulgar - Padrões de qualidade dos cuidados de enfermagem: Enquadramento
conceptual. Enunciados descritivos.

[2] World Health Organisation (WHO) (1986). Ottawa charter for health promotion.

[3] Kristenson M (2012). Impact of socioeconomic determinants on psychosocial factors and
lifestyle - implications for health service The Swedish experience. Soc Sci Med.

[4] Sánchez-Johnsen LAP, Anderson NB (2007). Health promotion and disease prevention. Encyclopedia of Health and Behavior.

[5] Keleher H, Parker R (2013). Health promotion by primary care nurses in Australian general
practice. Collegian.

[6] Diário da República (2014). Lei n. 21 de 16 de abril de 2014 (PT). Dispõe sobre a investigação
clínica.

[7] Borges TP, Kurebayashi LFS, Silva MJP (2014). Lombalgia ocupacional em trabalhadores de enfermagem massagem versus
dor. Rev Esc Enferm USP.

[8] Keleher H, Joyce CM, Parker R, Piterman L (2007). Practice nurses in Australia current issues and future
directions. Med J Aust.

[9] Alfaro-Lefevre R (2005). Aplicação do processo de enfermagem: promoção do cuidado
colaborativo.

[10] Lau DH (2002). Patient empowerment- a patient-centred approach to improve
care. Hong Kong Med J.

[11] Shoqirat N, Cameron S (2012). Promoting hospital patients' health in Jordan rhetoric and reality of
nurses' roles. Int J Nurs.

[12] Shoqirat N. (2014). 'Let other people do it...': the role of emergency department nurses
in health promotion. J Clin Nurs.

[13] Aguiar ASC, Mariano MR, Almeida LS, Cardoso MVLML, Pagliuca LMF, Rebouças CBA (2012). Percepção do enfermeiro sobre promoção da saúde na unidade de terapia
intensiva. Rev Esc Enferm USP.

[14] World Health Organisation (1997). The Jakarta declaration on leading health promotion into the 21st
century. Health Promot Int.

[15] Dudeja LCP, Singh A, Jindal AKY (2014). Health promotion initiatives An experience of a well women's
clinic. Med J Armed Forces India.

[16] Behma L, Wilhelmson K, Falk K, Eklund K, Zidén L, Dahlin-Ivanoff S (2014). Positive health outcomes following health-promoting and
disease-preventive interventions for independent very old persons Long-term
results of the three-armed RCT elderly persons in the risk zone. Arch Gerontol Geriatr.

[17] Yao X, Dembe AE, Wickizer T, Lu B (2015). Does time pressure create barriers for people to receive preventive
health services. Prev Med.

[18] Wang J, Chen CY, Lai LJ, Chen ML, Chen MY (2014). The effectiveness of a community-based health promotion program for
rural elders A quasi-experimental design. Appl Nurs Res.

[19] Lawless A, Freeman T, Bentley M, Baum F, Jolley G (2014). Developing a good practice model to evaluate the effectiveness of
comprehensive primary health care in local communities. BMC Fam Pract.

[20] Morales-Asencio JM, Martin-Santos FJ, Kaknani S, Morilla-Herrera JC, Fernández-Gallego MC, García-Mayor S (2014). Living with chronicity and complexity: Lessons for redesigning case
management from patients' life stories -A qualitative study. J Eval Clin Pract.

[21] March S, Torres E, Ramos M, Ripoll J, García A, Bulilete O (2015). Adult community health-promoting interventions in primary health care
A systematic review. Prev Med.

[22] Aldossary A, Barriball L, While A (2012). The perceived health promotion practice of nurses in Saudi
Arabia. Health Promot Int.

